# Hydrochlorate of Ammonia

**Published:** 1873-07

**Authors:** K. P. Moore

**Affiliations:** Knoxville, Crawford County, Ga.


### HYDROCHLORATE OF AMMONIA.

BY K. P. MOORE, M.D., KNOXVILLE, CRAWFORD COUNTY, GA.

[Read Before the Georgia Medical Association, April, 1873.]

My object in presenting this paper to the Association is not to cull from any authority save that of *experience* in every-day practice; hence my paper might properly be styled *Practical Observations* in the Use of Muriate of Ammonia. A few quotations to assist me in the explanation of the *rationale* of its action will be allowed.

"It acts primarily upon the alimentary canal;" is said by authors to purge in large doses, and to be constipating in small ones, but I have never observed these effects. "Secondarily it acts as a stimulating alterative upon the capillary, glandular, and lymphatic systems, etc., on the mucous, serous, and fibrous tissues, the nutrition of which it is supposed to improve."

It is also a very powerful defibrinating agent, and perhaps the most powerful resolvent in the whole materia medica.

I have never noticed any effects upon the action of the heart; indeed I do not believe that it either increases or decreases its action, primarily.

### PERTUSSIS.

We have, perhaps, no remedy in the materia medica, in the treatment of that peculiarly harrassing disease, pertussis, which affords more gratifying results. I claim in this use of the drug no originality, my attention being first directed to its use in this direction by Dr. H. V. M. Miller, while attending his clinic five years ago, since which time I have given it a fair test, and can verify the high encomiums bestowed upon it by that distinguished gentleman. In the spring of 1872 I had opportunity of giving it an extensive trial: my field of work was visited by a wide-spread epidemic of this distressing affection. Scarcely a child for miles around escaped its clutches, and upon every side I was greeted by the harsh whooping of some dear little patient, as he clung to the nearest object upon which he could support himself through a paroxysm of cough. The pleasure with which I know the profession will hail the presentation of any remedy beneficial in this distressing affection, prompts me to bring forward my experience with it. During the prevalence of the epidemic above referred to, I prescribed it, I suppose, to not less than one hundred patients; a close observation of quite a number of them, and a general observation of all, fully convinced me that it was certainly a very valuable remedy. If commenced in time, I was usually enabled to terminate a case in two weeks. Of course this was not always the case, and in a very few instances I was disappointed in stopping a case short of its usual course. I did not lose a single case.

Usually prescribed it in the following:

℞.—Ammonia mur. ℥ ss.

Paregoric ℥ ij.

Syr. squills ℥ iij.

Aqua ℥ iv.

M. S.—Teaspoonful *pro re nata*, regulated according to age, etc., of patient. I may remark here that it may be given *ad libitum.*

AMENORRHŒA, STERILITY, ETC.

I come now to notice a class of diseases in the treatment of which I claim the use of this drug to be purely original with me, viz: amenorrhœa and kindred troubles. I claim, also, an idea which, though it may not yet be fully established, and the *modus operandi* of its action not fully understood, yet it is one well worthy further investigation, viz: that this drug possesses some peculiarly specific action in stimulating healthy ovulation and its concomitant menstruation. Why should it not have this specific action? We have remedies which stimulate the liver to healthy action, remedies which act upon the kidneys, remedies whose specific action is upon the heart, etc. Now, as mercury, which, while it acts upon the glandular system and secreting organs generally, also possesses, at the same time, some peculiarly specific action upon the liver, why may not the ammonia—while it acts upon the glandular, mucous, serous and fibrous tissues (all of which enter largely into the composition of the menstruating organs) generally—also possess some peculiarly specific action in stimulating these organs to a healthy performance of their functions, a failure of which is too often the cause of ill health and heirless households? It may be claimed that the use of it in amenorrhœa is suggested by recent authors. So I admit, but at the date of the beginning of my experiments with it, I had never seen or heard of its being recommended; and *now*, as a cure for sterility, and as a stimulant to healthy ovulation, I have seen on record no experiments as satisfactory as those I have been prosecuting, a few of which I propose to bring forward.

It was by accident I was led to experiment with it in this direction. In 1868, I was treating a friend of mine for ovarian cysts; was prescribing hydrochloras ammoniæ as a stimulating alterative upon the diseased organ. The effect of bringing on and regulating her menses was so magical, she was induced, without my knowledge or consent, to give it to her daughter, aged fourteen, who had never become regular in her catamenial function. Subsequently she gave it to her cook, who was also irregular, and, in both cases it acted so kindly that she called my attention to it. It was something new to me, but thinking it worthy a trial, I afterward prescribed it in quite a number of cases with very gratifying results.

In 1869, was consulted by an intelligent negro for a prescription for his wife, aged about twenty-eight, who was irregular. She had been married nine or ten years, and had never borne children; was thought by her owners to be sterile. I gave her twenty grains mur. ammonia, ter die, for twelve days preceding her expected period. Continued it for several months. Nothing could have acted better. In about twelve months from the beginning of prescription she gave birth to a fine son, much to the joy of the parents. She now has her second.

May 25th, 1873, prescribed for Betty (colored), aged fifty, mother of several children, the youngest now seventeen years old. After getting a full history of her case, I concluded that she was suffering with the usual symptoms accompanying the "critical period," as it is called, though later than the average age in life. By way of experiment, I gave her mur. ammonia.

For several months I lost sight of her. At last I was called to see her. Found her suffering with "morning sickness" and the usual signs of pregnancy, and ventured to express my suspicions. She could not believe it, "seeing she was well stricken in years," and her youngest now seventeen years old. I made vaginal examination, and found uterus about size of three month's gestation, and being so thoroughly satisfied with my diagnosis, I used no remedy only such as to give tone to general health. Was with her at confinement, and delivered her of a delicate female child.

July 6th, 1870, prescribed for negress, aged about forty, mother of several children, youngest five or six years old. Irregular and deficient menses. Gave the ammonia as in preceding cases.

Called in less than twelve months from date of prescription to attend her in accouchment; she was greatly displeased that I "had started her to breeding."

These cases, taken from several that have come under my observation, certainly seem to invite attention in this direction to the virtues of muriate of ammonia. They served to get up for me an unenviable reputation; for I have been consulted and tantalized by every man in the whole country whose wife gave him no heir, for some of that "breeding medicine."

Now, while I do not, of course, believe this remedy to possess any virtue in causing conception, other than a stimulation to healthy ovulation and menstruation, yet in this way I am greatly inclined to believe it worthy of notice, to say the least of it. In one case in which I was giving it, as uterine and general alterative, about the "turn of life," she told me that it produced such a free flow of the catamenia as to cause alarm; she sat for hours where she could allow it to drop in a night-glass, and it kept a constant continual dripping for hours. To give it further trial, I advised it to be continued at the next period as before,—that is, begin twelve days before, etc. The effect was the same, and I abandoned it.

In a few exceptional cases, I have been disappointed with it, and in *one* which comes to mind just now, it rather seemed to lessen the flow. Trial and expression from the members is earnestly desired.

ENLARGED GLANDS, LIVER, SPEEN, ETC.

In almost all glandular affections, especially fibrinous deposits, growths, etc., I have found the ammonia highly serviceable. In my section, where malaria runs high at certain seasons of the year, I have frequently enlarged livers, spleens, etc., and my favorite prescription is muriate ammonia, with topical application of tincture of iodine over the surface, and *very* rarely ever have to resort to anything else. The *rationale* of its action in these troubles I will more fully explain further on, when I come to notice its use in valvular heart troubles, hemicrania, etc.

INTERMITTENT FEVER.

I have, in quite a number of cases, given it with success in intermittent fever. During the malarious season last year, I put it to the test, and as said above, was successful in a large per cent; but in a large majority I was obliged to call the quinine to my aid, though I think it made the amount of quinine necessary less than if the ammonia had not been used. "In 1851, Dr. Aran reported his success with this remedy in the treatment of intermittent fever, to the Academy of Medicine, Paris, having cured eleven out of thirteen cases." It is, at least, worth a trial with those who have to do a large and unreliable practice in the malarious sections, and furnish their own quinia at from three dollars to four dollars per ounce. In bilious colic, facial neuralgia, rheumatism, etc., it acts finely.

VALVULAR HEART DISEASE, HEMICRANIA, ETC.

For fear that this paper may carry with it the semblance of empiricism, I will endeavor, in presenting its use in the above terrible troubles, to give the rationale, not only of the diseases under this head, but in so doing, also cast light back over the affections already passed under review.

During 1872, saw Susan Barber (colored), aged eighteen, suffering with considerable dyspnœa, and severe pain in præcordial region. Auscultation revealed considerable hypertrophy, with excessive labored action of the heart, more or less dropsy, especially of the lower extremities, very great cooing, bellows-like regurgitating sound, so very characteristic of valvular deficiency. Gave tr. digitalis, mercury, etc., (all the usual remedies) with no benefit at all. No excitement, either mental or physical, could be at all tolerated without producing the most distressing symptoms. My patient grew from bad to worse, and I expected she would die. Finally, I reasoned in this way: I have here valvular trouble, perhaps fibroid growth about the semilunar valve, or the sinuses of Valsalva, or like fibrinous deposit upon the columnæ carnæa, caught there in the same way that fibrin is collected by whipping straws through blood drawn from a vein. The primary cause may have been, possibly, some slight inflammatory exudation, however slight at first, yet it formed a nucleus around which might have been collected this fibrin. This fibroid deposit continued to grow; the natural passages through the heart becoming obstructed, greater effort was necessary on the part of the heart to carry its load of blood through its rounds, and by its over-exertion it becomes hypertrophied, and, as all understand, dropsy is the consequence. Now, it has been demonstrated that coagulated fibrin can readily be dissolved by the addition of muriate of ammonia; it is, therefore, a very rational expectation that we may hope to cure this patient, if the condition of things exists which we have supposed. I determined, therefore, to make the experiment; hence I gave her mur. ammo. grs. 25 to 30 ter die, continued also tr. digitalis. As soon as my patient's blood became charged with the ammonia, it began to dissolve the fibrin, and gradually it wasted away. The obstruction being removed, the force of the heart's action became less, the hypertrophy gradually disappeared, and the patient's general condition improved. At present she can run, jump, and dance half the night.

It will be seen that I do not mean by this to include all valvular diseases, but only such as are of a fibrinous character.

In 1868, was called to see S. C., aged five years; got following history: Five weeks before I saw her, she was seized with a very hard convulsion, in which all the muscular system was thoroughly drawn, especially those of the neck, from which time she was a distressing victim of hemicrania. For five weeks she had never been out of the nurse's arms—neck drawn around to one side, continued biting shoulder, had been reduced to a perfect skeleton, and was truly an object of pity.

I was impressed with the following suspicions: While convulsion was on, the jugulars were so compressed by the sterno-mastoid muscles as to prevent the return of blood from the brain; the arteries being deeper seated, the blood was pumped into the cerebral circulation, distending the blood vessels until one was ruptured, allowing extravasation, and a consequent compression from clot of blood. Knowing the powers of ammonia to dissolve coagulated blood, I prescribed it in large doses. In from ten to twenty days my patient began to improve rapidly, and was soon quite well.

